# Supplementary material for: Situational assessment of empathy and compassion: Predicting prosociality using a video-based task
Source: PLoS One. 2023 Dec 7;18(12):e0289465. doi: 10.1371/journal.pone.0289465 (PMC10703325; doi:10.1371/journal.pone.0289465)
Supplement: S1 File — (DOCX) [file pone.0289465.s001.docx]

**SUPPLEMENTARY MATERIALS** for the manuscript:

Situational Assessment of Empathy and Compassion: Predicting prosociality using a video-based task

Authors: Gabriela Górska, Aviva Berkovich-Ohana, Olga Klimecki, Fynn-Mathis Trautwein

**Supplement A**

Pearson’s correlation tests for the SoVT compassion rating and the Compassionate-Love Scale (CLS), Empathic Concern subscale of the Interpersonal Reactivity Index (IRI) and the hypothetical helping questions measure (HHQ), with the FDR correction for multiple comparisons.

|  | *rₚ* | *p_uncor._* | *p_FDR_* | *n* |
| --- | --- | --- | --- | --- |
| CLS | .35 | .00 | .005 | 78 |
| EC(IRI) | .24 | .04 | .05 | 78 |
| HHQ | .20 | .08 | .08 | 78 |

**Supplement B:**

T.test results of the pilot study: for compassion where LE (*M* = 5.51, *SD* = 1.58) and HE videos (*M* = 1.78, *SD* = 2.34) were compared, *t*(19) = 4.57, *p* < .01, and for negative affect (LE: *M* = 1.28, *SD* = 1.13; HE, *M* = 4.72, *SD* = 2.37), *t*(16) = 4.53, *p* < .01.

**Supplement C:**

The script of the real-life helping question:

*Before we start with the study, we would like to invite you for a wonderful event. [Our] Lab is preparing a special event for high-school-aged children with fetal alcohol spectrum disorder. It’s a disorder caused by alcohol use during pregnancy that can severely and negatively influence the appearance, cognitive skills and emotional wellbeing of a child. They often need some support in the education process, and as a part of the Center for Learning Disabilities, we are organizing a project to support children with this kind of life challenge. We have children involved in the project within the whole country! If you feel like supporting this project, please let us know in which of the activities below you can get involved! It’s on a fully voluntary basis, and by the end of the semester we will contact only those who agreed to help to follow up.*

*You can (please, indicate 0, or more options):*

*a) promote the event by sharing it on Facebook;*

*b) support us with a short amount of time for administrative work (correct pages of 10-15 materials written in Hebrew)*

*c) take part in a 1 day long fest, the time and space to be agreed on;*

*d) give a private class to one child from the project once a week for two months, the exact time and space to be agreed on.*

**Supplement D:**

The conflict-related questions:

a) on issues related to the tensions Israel has with other countries/societies, to what extent do you support more aggressive actions initiated by Israel, or more conciliatory ones? (scale: 1 - aggressive actions to 10 - conciliatory policies); b) Negative emotions to the neighbouring nations to your country ("hostility", "anger", "hate"), (scale: 1(did not feel like it at all) to 4 (I felt a lot of this emotion)); c) Perceived threat from the neighbouring nations to your country (“they proved constantly that they are unreliable, and are able to turn their backs on Israel at any time”; “they are a threat to the security of Israel”; “The different lifestyle of them endangers Israeli culture”; “They are a persisting threat to the existence of the State of Israel”). (scale: 1 (I do not support this statement at all) - 4 (I support the statement strongly)) d) Negative emotions to the Israelis supporting the opposite policies than you ("hostility", "anger", "hate"), (scale: 1(did not feel like it at all) -4 (I felt a lot of this emotion)); e) Perceived threat from the Israelis supporting the opposite policies than you. (“they proved constantly that they are unreliable, and are able to turn their backs on Israel at any time”; “they are a threat to the security of Israel”; “The different lifestyle of them endangers Israeli culture”; “They are a persisting threat to the existence of the State of Israel”). (scale: 1 (I do not support this statement at all) - 4 (I support the statement strongly)).

**Supplement E:**

Vladimir’s Choice (Sidanius et al., 2007) description:

Participants could choose between pairs of scholarships (respectively), for example the 1^st^ option with 17 scholarships (for the best students) vs. 20 scholarships (for Palestinian students). This option gives the total gain of 37 scholarships, with the biggest *maximum gain* of all the options. However, for a person following the *maximum difference* principle, the “outgroup” - Palestinian students - would receive more (20) than the “ingroup” (17), hence it is an uneven option. Other possible options were: (2^nd^)15 vs.16, (3^rd^) 12 vs.13, (4^th^) 10 vs.10, (5^th^) 9 vs. 8, (6^th^) 7 vs. 6, (7^th^) 6 vs. 2. The last one is of maximum difference: even though both groups receive the least, the ingroup would receive much more in proportion to the outgroup.
